# Home-based record (HBR) ownership and use of HBR recording fields in selected Kenyan communities: Results from the Kenya Missed Opportunities for Vaccination Assessment

**DOI:** 10.1371/journal.pone.0201538

**Published:** 2018-08-02

**Authors:** David W. Brown, Collins Tabu, Kibet Sergon, Stephanie Shendale, Isaac Mugoya, Zorodzai Machekanyanga, Peter Okoth, Iheoma Ukachi Onuekwusi, Ikechukwu Udo Ogbuanu

**Affiliations:** 1 Brown Consulting Group International LLC, Cornelius, North Carolina, United States of America; 2 Kenya Expanded Programme on Immunization, Nairobi, Kenya; 3 World Health Organization Kenya Country Office, Nairobi, Kenya; 4 World Health Organization Headquarters, Geneva, Switzerland; 5 Maternal and Child Survival Program, Nairobi, Kenya; 6 World Health Organization Regional Office for Africa Inter-Country Support Team, Harare, Zimbabwe; 7 UNICEF Kenya Country Office, Nairobi, Kenya; University of Texas Medical Branch at Galveston, UNITED STATES

## Abstract

**Background:**

Home-based records (HBRs), which take many forms, serve as an important tool for frontline health workers by providing a standardized patient history vital to making informed decisions about the need for immunization services. There are increasing concerns around HBRs with recording areas that are functionally irrelevant because records are incomplete or not up-to-date. The aim of this report was to describe HBR ownership and report on the utilization of selected recording areas in HBRs across selected study communities in Kenya.

**Methods:**

The Kenya Missed Opportunities for Vaccination Assessment utilized a mixed-methods approach that included exit interviews, using a standardized questionnaire, among a convenience sample of caregivers of children aged <24 months attending a health facility during November 2016 as well as interviews of health staff and facility administrators. In addition to the exit interview data, we analysed data obtained from a review of available HBRs from the children.

**Results:**

A total of 677 children were identified with a valid date of birth and who were aged <24 months. A HBR was in hand and reviewed for three-quarters of the children. Nearly one-third (n = 41) of those without a HBR in hand at the visit noted that they did not know the importance of bringing the document with them. Roughly two-thirds (n = 443) of caregivers noted they were asked by clinic staff to see the HBR during the clinic visit. Across the 516 reviewed HBRs, recording areas were most commonly identified for the child’s demographic information (80% of HBRs) and vaccination history (82%) with information marked in >90% of records. Recording areas were less frequently available for child early eye / vision problems (61%), growth monitoring (74%) and vitamin A (76%); with information marked in 33%, 88% and 60% of records, respectively.

**Conclusions:**

Critical to the reduction of missed opportunities for vaccination, the HBR’s importance must be emphasized and the document must be requested by health workers at every health encounter. Health workers must not only ensure that all children receive a HBR and counsel caregivers of its importance, but they must also ensure that all sections of the record are legibly completed to ensure continuity of care. Programmes are encouraged to periodically review and critically assess the HBR to determine whether the document’s design and content areas are optimal to end user needs.

## Background

Home-based, personal health records, such as vaccination cards or child health passports, are a cost-effective tool designed to provide frontline health workers with a standardized patient history that is convenient, comprehensive and vital to making informed decisions about the need for care and immunization services [1]. In many low- and middle-income countries, home-based records (HBRs) complement facility-based record systems and serve as a verified surrogate in the absence of functioning facility-based record systems. Beyond these critical roles as a medical record, HBRs also serve as a prompt to initiate a discussion between health workers and caregivers about the importance of immunization during a health encounter at a facility or during an outreach session. The HBR extends the relationship between the health worker and client beyond an individual health encounter, reinforcing the importance of the HBRs and perhaps increasing the likelihood of a timely completion of the full infant immunization series. As a source of documented evidence of vaccination history, HBRs are also important for public health monitoring, improving the accuracy and reliability of small-scale community-based rapid coverage assessments (i.e., 75-household surveys [2]), population-based cluster coverage surveys [3] and studies of missed opportunities for vaccination (MOV) (defined as any contact with health services by an individual who is eligible for vaccination (e.g. unvaccinated or partially vaccinated and free of contraindications to vaccination), that does not result in the person receiving one or more of the vaccine doses for which they are eligible [4]).

In order to fulfil their functional purpose, HBRs must be available in the right place, at the right time and in the right quantity to be distributed [5,6] and must be valued and retained by caregivers [7]. Caregivers must bring the HBR to each health contact with the child. Health workers at each point-of-service must fill in and update the HBR to maintain a complete, up-to-date health history. Illegible or improperly completed records and lost or damaged records undermine efforts to maintain and improve continuity of care across health encounters. When health workers regularly reference and utilize the HBR, this is likely to convey the value of the HBR to caregivers. Conversely, an unmarked or incomplete HBR may inadvertently communicate to caregivers that the HBR, or a particular recording area on the HBR, is not important.

Home-based records take many forms, from those focused on recording infant vaccination history only to more comprehensive child and maternal health books [8]. There are increasing concerns that many of the recording areas in HBRs, particularly in more comprehensive child and maternal health books and vaccination plus growth monitoring records, are functionally irrelevant because records are incomplete or not up-to-date. These concerns arise due to increasing pressures on programmes to ensure adequate supplies of HBRs for the annual birth cohort and the fact that more comprehensive HBRs are inherently more expensive per unit to print than simple vaccination only records [8]. Moreover, there is an awareness of the potential indirect negative messaging that may be sent to caregivers about the importance (or lack thereof) of certain child survival domains if the recording areas for that domain are left incomplete in the HBR.

There are few studies that have examined completeness of HBRs. A study conducted in South Africa of the completeness of Road-to-Health Books within the first six weeks of life across three domains (infant birth weight, BCG vaccination, maternal syphilis results) observed that less than half of the children’s HBRs were observed to be complete [9]. Recent anecdotal reports and informal HBR reviews [10] have highlighted that demographic/background characteristics and vaccination history are often the only recording fields in the HBR regularly completed by health workers. Other recording areas remain blank or incomplete. Similar observations were made in a review of HBRs belonging to children aged two to twelve months attending a maternal and child health clinic in Mbagathi District, Kenya [11].

As part of a series of activities by the World Health Organization (WHO) and the United Nations Children’s Fund (UNICEF) to revitalize the utilisation of HBRs within immunization service delivery (and primary health care more broadly), we leveraged an opportunity within the 2016 Kenya Missed Opportunities for Vaccination Assessment to describe current HBR ownership prevalence among children visiting health clinics and report on the utilization of selected recording areas in HBRs in Kenya.

## Methods

### Study setting

This study is a secondary analysis of data collected as part of a WHO-led initiative, conducted in collaboration with government programme officials and immunization partners in Kenya, to characterize MOV among children aged less than two years. The MOV assessment is designed to be completed in less than 10 days inclusive of training, data collection and preliminary data analysis. It is a mixed-methods approach that consists of exit interviews with caregivers attending a health facility, knowledge-attitudes-practices surveys of health workers, in-depth interviews of health administrators and semi-structured, qualitative interviews with health workers and caregivers. The assessment which provided the data for this secondary analysis was conducted during November 2016 using revised methods [12] based on assessments conducted in 1988 [13] and 2013.

### Selection of health facilities

The Kenya MOV assessment team selected 40 health facilities across 10 counties (4 health facilities per county). Counties were selected purposively to represent a range of geographic areas and immunization performance levels, which were based on administrative vaccination coverage for the third dose of diphtheria-tetanus toxoid-pertussis-hepatitis B-*Haemophilus influenzae* type b (or pentavalent) vaccine. The sampling of health facilities was guided by a WHO recommendation to assess at least 30 health facilities, when possible, with a minimum of 20 health facilities to be visited. Health facilities were selected to reflect a range of sizes (i.e., Kenya Essential Health Package levels 2–5), types (Ministry of Health, nongovernmental organization, religious, private), and locations (urban and rural). Because of restrictions on the number of days available for data collection, logistical access of selected areas was also considered.

### Study field activity

Prior to going to the field, the study team reviewed and customized a generic questionnaire [14] to align with the specific vaccination schedule and terminology used in Kenya. Interviewers and supervisors were drawn from Kenya Ministry of Health staff, WHO/Kenya, WHO Regional Intercountry Support Team for East and Southern Africa, UNICEF/Kenya, Clinton Health Access Initiative, WHO Headquarters, United States Centers for Disease Control and Prevention and the United States Agency for International Development sponsored Maternal and Child Survival Program. All interviewers were centrally trained between 31 October and 2 November 2016 in Nairobi. A pilot test of the questionnaire was carried out during a half-day field exercise included in the training, during which interviewers went to five different health facilities in Nairobi to practice conducting interviews.

Ten field teams were formed (one team per county) to conduct a minimum of 600 exit interviews with caregivers during a three- to five-day period. Each team was expected to complete at least 20 sequential exit interviews with caregivers per day. Field teams were comprised of two to three interviewers (25 interviewers total) and overseen by a supervisor (three supervisors in total, each responsible for three or four teams). Each team and each supervisor was supported by a driver (13 drivers total). A target sample of 300 health workers (10 health worker interviews per health facility) was also interviewed to gather information about reasons for MOV; however, these are not the focus of this manuscript. All interviews in the Kenya MOV assessment were conducted during 3–8 November 2016.

Interviewers were instructed to position themselves at the exit or other strategic location of selected health facilities. All caregivers of children age 0–23 months exiting selected health facilities were eligible to be interviewed regardless of the reason for visiting the health facility, their place of residence or relationship to the child. For this study, the caregiver was defined as the person accompanying the child at the time of the interview and may have been the person who gave birth to or adopted the child or was otherwise taking care of the child, such as an aunt, grandmother, or father. All persons exiting the health facility with a child were sequentially approached and asked to participate in the study. Each potential participant was pre-screened on age of accompanying child only. Per protocol, exit interviews were conducted with adult caregivers (≥15 years of age) accompanying children between the ages of 0–23 months visiting one of the study selected health facilities on the day of the assessment.

Prior to each interview, the selected individual was made aware that their participation was voluntary and they were asked to provide verbal consent. Caregiver exit interviews were conducted by trained interviewers in the appropriate local language. If a consenting adult caregiver was accompanied by more than one child, the interviewer was instructed to focus the exit interview on the youngest child. All consenting adults were interviewed irrespective of the availability of a HBR at the time of the interview. For children without a HBR, teams abstracted dates of vaccination from health facility registers after completing caregiver interviews.

Field interview teams were instructed to make an effort to identify a mix of caregivers with infants (aged 0–11 months) and one-year old (12–23 months) children, and if possible, to conduct 10 interviews with caregivers of children in each age group at each selected facility. Field teams were also asked to conduct interviews at health facilities on days and during hours (usually morning hours) when immunization services were occurring, and to interview the caregivers *after* they had received service at the facility. Field teams were also instructed to interview consecutive eligible caregivers at the exit of the health facility, so as to achieve a mix of caregivers attending the facility for a variety of purposes (i.e. immunization as well as other services). All data was collected electronically using tablets programmed with the standardized exit interview questionnaire.

As part of each exit interview, caregivers were asked several questions related to how they obtained and used their child’s HBR (see S1 Appendix). Additionally, among caregivers with a HBR in hand, the field team reviewed the recording areas that appeared on the HBR. Specifically, the teams identified whether the following sections existed on the child’s HBR: background demographic information, vaccination history, receipt of vitamin A, growth monitoring, early eye or vision screening and newborn delivery information. If a recording field existed, the team also noted if an effort had been made to record information in the section. A recording field was deemed filled or marked if ANY deliberate entry was observed in the recording area.

### Data analysis

The aim of the WHO MOV assessments is to provide a national immunization programmes with a rapid, snapshot characterization of missed opportunities in selected areas. The pooled data obtained from the purposive sampling of health facilities and non-random, sequential convenience sample of caregivers (and their children) were analysed using simple descriptive summary statistics. All analyses were conducted using Stata v14 (Stata Corporation, College Station, Texas).

### Ethical considerations

The Missed Opportunities for Vaccination protocol was submitted to the Kenyan Ministry of Health for ethical review and was deemed a Government of Kenya led programme assessment, and was therefore exempt from further review.

## Results

A total of 690 children were identified exiting a clinic with an adult. This analysis uses the data obtained from 677 children with a valid date of birth and who were aged <24 months. Among the 13 children who were excluded from the analysis, five mothers reported no formal education, two mothers reported some education but did not complete primary school, three mothers reported completing secondary school and information was unavailable for three mothers. Nine of the 13 children reportedly had received at least one vaccination in their lifetime (data not available for two children), and seven of the 13 children had a HBR with them at the visit (data not available for two children).

Among the 677 children with a valid date of birth and aged <24 months, the underlying reason for visiting the clinic included medical consultation or routine visit (n = 372); vaccination (n = 187), hospitalization of the child (n = 9) and other reasons (n = 11; missing data for reason for visit, n = 13). In 85 instances, the child was accompanying their caregiver to the clinic, as opposed to seeking services. A summary of background characteristics for the children and their respondent caregivers is provided in Table 1.

**Table 1:** Summary of background characteristics of study target children and their mothers: Kenya Missed Opportunities for Vaccination Assessment, 2016.

	n (%)
**CHILD CHARACTERISTICS**	
Child Age	
< 6 months	261 (39)
6–11 months	208 (31)
12–23 months	188 (28)
Unknown	20 (3)
Child Sex	
Male	338 (50)
Female	331 (49)
Missing	8 (1)
**RESPONDENT CHARACTERISTICS**	
Relationship to child	
Mother	638 (94)
Father	5 (1)
Sibling	5 (1)
Other relative	14 (2)
Missing	15 (2)
Formal education level	
None	65 (10)
Some primary education	95 (14)
Completed primary education	280 (41)
Completed secondary education	159 (23)
More than secondary education	74 (11)
Missing	4 (1)
Occupation	
Housewife	319 (47)
Employee	89 (13)
Farming	91 (13)
Self-employment—boss /employer	151 (22)
Student in school	24 (4)
Missing	3 (<1)
Person who decides to vaccinate child	
Father	24 (4)
Mother	302 (45)
Other relative	14 (2)
Consensus of mother and father	330 (49)
Other	1 (<1)
Missing	6 (1)
County of health facility	
Bungoma	80 (12)
Kajiado	36 (5)
Kiambu	59 (9)
Kitui	44 (6)
Migori	74 (11)
Mombassa	83 (12)
Nakuru	64 (9)
Taita Taveta	83 (12)
Trans-Nzoia	80 (12)
West Pokot	74 (11)

A HBR was in hand and available for review for three-quarters (n = 516; current HBR ownership prevalence = 76%) of children (Table 2). Nearly 20% (n = 129) of respondents noted they owned a HBR for the child but did not have the document with them. Among the 129 respondents that did not have the child’s HBR in hand, nearly one-third (n = 41) reported they did not come to the clinic for immunization services. Similarly, nearly one-third (n = 41) of those without a HBR in hand at the visit noted that they did not know the importance of bringing the document with them. Four percent of respondents reported not having a HBR at all. Reasons for not having a HBR included having never received a HBR (n = 9), having lost the document (n = 4) and other reasons (n = 12) (NB. data were missing for 4 respondents who reported not having a HBR).

**Table 2:** Responses to home-based record-related questions by 677 caregivers of children aged <24 months visiting a primary health care clinic: Kenya Missed Opportunities for Vaccination Assessment, 2016.

	N (%)
Does your child have a vaccination card / health passport?	
Yes, and I have it with me	516 (76)
Yes, but I do not have it with me	129 (19)
No	29 (4)
Missing	3 (<1)
Ever lost a vaccination card / health passport for this child?	
Yes	40 (6)
No	596 (88)
Missing	41 (6)
Purpose the vaccination card / health passport serves (*respondent may select more than one response)*	
To know what vaccines the child has received and which are missing	444 (65)
Birth certificate and/or identification	24 (3)
Overall health record and growth monitoring	77 (11)
Record and remind for return visit dates	25 (4)
Other	51 (7)
Don’t know	57 (8)
Missing	33 (5)
During today’s clinic visit, did the staff ask you for the child’s vaccination card / health passport?	
Yes	443 (65)
No	230 (34)
Missing	4 (1)
Have you ever been asked to pay for a health card / passport for your child?	
Yes	113 (17)
No	554 (82)
Missing	10 (1)
What type of health facility asked you to pay? (asked only if response ‘Yes’ to question above)	
Public	81 (72)
Private	27 (24)
Missing	5 (4)

**Fig 1:**
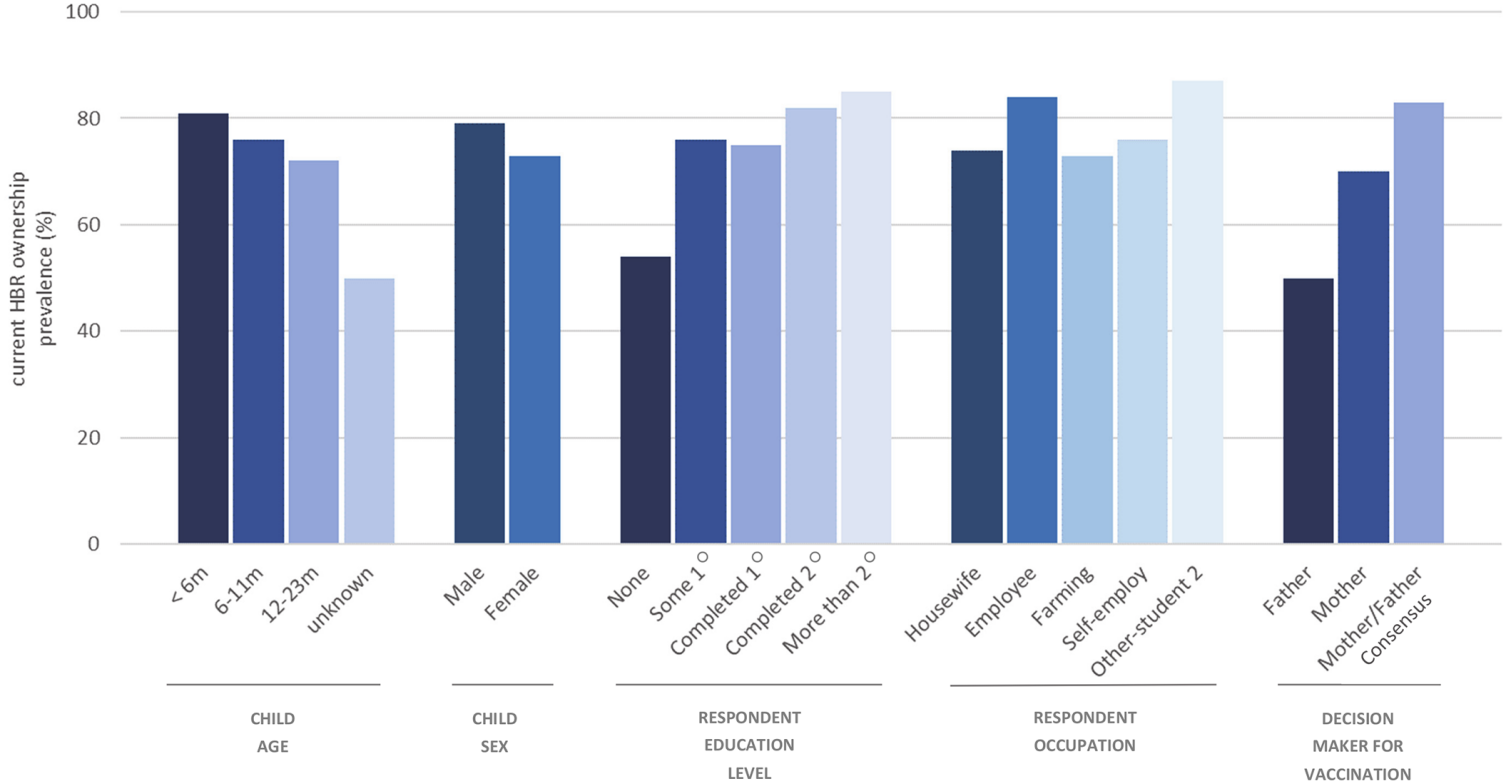
Current HBR ownership prevalence among 677 children aged <24 months by selected background characteristics.

Current HBR ownership prevalence was greater among children aged <6 months than those aged 12–23 months (81% vs 72%) and was lower for those where the respondent reported no formal education (54%) compared to respondents who completed secondary or more than secondary schooling (82–85%) (Fig 1). Current HBR ownership levels were also greater among those where decisions about vaccination were a shared responsibility of the mother and father (83%) than by either the father (50%) or mother (70%) alone.

When asked to select the purpose of HBRs, nearly two-thirds (n = 444) of respondents identified HBRs as a tool for determining which vaccines the child had received and which were missing (Table 2). Fewer than 10% reported not knowing the purpose of the HBR. Other perceived HBR purposes included the role of the HBR as a record of care and growth monitoring (n = 77), as a tool for accessing treatment and clinic services (n = 31) and as a reference for the return date for vaccination (n = 25). Less commonly, respondents noted the importance of the document for obtaining a national identification number and birth certificate (n = 24) or for school entry (n = 6).

Six percent (n = 40) of respondents noted that they had lost their child’s HBR at least once in the past; more than half (n = 27) of these reported no difficulty replacing the document. Nearly 20% (n = 113) of respondents reported that they had been asked to pay for their child’s HBR at some point in the past, most frequently by staff at public health facilities.

About two-thirds (n = 443) of respondents noted they were asked by clinic staff to see the HBR during the clinic visit. Most (411 of 443, or 93%) respondents asked to show the HBR during the visit had the HBR in hand (HBR forgotten, n = 23; no HBR ever, n = 6; missing, n = 3). Among the 184 children vaccinated during the clinic visit, nearly all (99%; n = 182) respondents noted that the health worker asked to see the HBR and most of these (97%; n = 178) had the record in hand; in contrast, only 53% (n = 259) of caregivers of the 484 children who did not receive vaccination that day were asked to view the HBR (HBR in hand, n = 232; HBR forgotten, n = 19; no HBR, n = 5; missing data, n = 3).

Interviewers reviewed 516 HBRs to determine whether six recording areas were included in the HBR and whether information had been marked or notated in those sections (Table 3). Recording areas most commonly existed for the child’s demographic and background information (e.g., name, sex, date of birth) and vaccination history (80% and 82%, respectively) with information marked in these areas in more than 90% of records. Recording areas were less frequently available for child early eye / vision problems (61%), growth monitoring (74%) and vitamin A (76%); with entries logged in these fields for 33%, 88% and 60% of records, respectively.

**Table 3:** Availability of selected recording areas in the home-based record and marked information in those recording areas among 516 reviewed home-based records: Kenya Missed Opportunities for Vaccination Assessment, 2016.

Recording area available for …		Information marked in HBR		
Child background				
Yes	413 (80%)	Marked?	Yes	384 (93%)
No	36 (7%)		Unsure	6 (1%)
missing	67 (13%)		Missing	23 (6%)
Child vaccination history				
Yes	422 (82%)	Marked?	Yes	395 (94%)
No	29 (6%)		Unsure	8 (2%)
Missing	65 (12%)		Missing	19 (4%)
Child vitamin A record				
Yes	392 (76%)	Marked?	Yes	263 (67%)
No	51 (10%)		Unsure	75 (19%)
missing	73 (14%)		Missing	54 (14%)
Child growth monitoring chart				
Yes	381 (74%)	Marked?	Yes	336 (88%)
No	56 (11%)		Unsure	24 (6%)
missing	79 (15%)		Missing	21 (6%)
Child newborn health / delivery				
Yes	339 (66%)	Marked?	Yes	205 (60%)
No	56 (11%)		Unsure	85 (25%)
missing	121 (23%)		Missing	49 (14%)
Child early eye / vision problems				
Yes	314 (61%)	Marked?	Yes	103 (33%)
No	84 (16%)		Unsure	141 (45%)
missing	118 (23%)		Missing	70 (22%)

## Discussion

In this study of children aged <24 months visiting a health facility in Kenya, 76% of children currently owned a HBR and brought the document with them to the clinic. Although the results of the study are not necessarily generalizable to the national population because of the non-random, sequential convenience sample of caregivers and their children and the deliberate sampling of health facilities, the observed current HBR ownership prevalence is consistent with the results of the 2014 Kenya Demographic and Health Survey wherein *ever* and *current* HBR ownership among children aged 12–23 months was 99% (95% confidence interval: 98, 99) and 75% (73, 77), respectively [15]. HBR ownership levels have been increasing over the prior decade in Kenya [15], though HBR loss rates (i.e., the relative difference between ever and current HBR ownership) remain high, near 25% (loss rate <10% is desired). Continued efforts are needed to ensure that durable HBRs are widely available in the right place, at the right time and in the right quantity in order to maintain high levels of HBR ownership [5,6,16].

In its most basic form, the HBR is a medical document on which all primary healthcare services received by an individual are recorded. As such, HBRs are critical in the reduction of missed opportunities for vaccination. When an individual comes to a health facility, for any reason, the health worker should ask for and review the HBR. After reviewing the health history in the HBR and cross-referencing the facility-based record(s) (a task often not possible in many low- and middle-income countries), the health worker updates any missing information in the HBR, provides necessary healthcare service(s) and carefully records the delivered service(s). Ideally, entries in the HBR are made in clear, legible handwriting along with the date of service, the date of the next expected visit and any additional information that may be useful for subsequent healthcare providers. Specific to immunization, the HBR plays a catalytic role in prompting health workers to review an individual’s vaccination history and discuss the importance of timely vaccination with caregivers, particularly during the first year of life. To facilitate this, caregivers must bring and health care workers must review the card at every visit.

The patterns we observed in HBR presentation at health facilities, reinforce the consequence of health workers acknowledging the HBR as an important document that should be brought to and reviewed at each-and-every health encounter, regardless of whether the visit is for immunization services. Across study sites, while nearly all children who received vaccinations during the clinic visit were asked for their HBR by health staff, only two-thirds of caregivers, overall, reported that they were asked to show their child’s HBR during the clinic visit. Among the children who were not vaccinated, roughly half were asked to show their HBR. Roughly one-third of caregivers in this study who did not bring their child’s HBR to clinic noted that they did not bring the document because they were not coming for immunization that day. Research has shown that important reasons for MOV is a caregiver being inadequately informed about the benefits of vaccines, recommended vaccination schedule and the child’s next vaccination visit [17]. If the HBR is simply left at home (but not permanently lost or damaged beyond repair), health workers should still provide documented evidence of which vaccination was received and the date of next vaccination alongside supportive messaging to caregivers to bring both the HBR and the temporary document to the next visit (the complete history should then be transcribed into the HBR at the next soonest opportunity).

More than 80% of caregivers provided valid explanations of the purpose of the HBR. Among the responses, 31 caregivers noted that the HBR is required, or was perceived by the respondent to be required, to access primary care services in some communities in Kenya. We do not believe that the absence of the HBR is appropriate justification for restricting access to care. Furthermore, despite explicit language printed on Kenya’s official HBR (since at least 2014) that the document is not for sale, nearly 20% of respondents had been asked to pay for a HBR, many by public health facilities. Prior work [18] has highlighted that although the HBR is officially provided free of charge, there may be a difference between policy and practice and health workers may use the HBR to generate small amounts of income [19]. Some caregivers may have received older versions of the Kenya HBR or received a private sector HBR that did not include explicit language printed on the document that the record is not for sale. In other cases, the HBR was an informal ruled-notebook, a makeshift solution resulting from a problem of stock outs of the official HBR in the country. Because private health facilities are often for-profit businesses, it is, perhaps, not unusual to find private health facilities charging for the HBR, particularly if the private facility maintains its own version rather than issuing the national or local government’s HBR version. Although there are some who support charging a nominal fee for the document to incentivize more careful ownership, we are unaware of any research supporting such a viewpoint. Programmes are encouraged to provide immunization services and HBRs (including replacement of those lost or damaged) to children free of charge [1].

The current version of the Kenya HBR, originally introduced in 2010 and revised several times since, is a comprehensive maternal and child health handbook (the record is available for viewing online [20]) that contains all six of the recording fields evaluated in this review. Older versions of Kenya’s HBR also include background demographics, vaccination history and growth monitoring recording areas. However, we observed that these recording fields did not consistently appear in the reviewed HBRs. This may suggest that a variety of different HBR forms exist in the field. A cursory review of electronic images taken of the HBRs in this project suggests that at least 10 different HBR formats were identified by the study field teams. This observation is consistent with a study in Viet Nam which highlighted the presence of multiple HBR versions and the challenges this presents for health workers [21]. Multiple HBR formats may also indicate fragility of the currently issued maternal and child health handbook which allows pages to be separated from the document. The use of multiple HBR formats can pose challenges for health workers, for example, making it more difficult to locate recording fields which may lead to incomplete or blank recording fields.

Our observations of the child survival related recording fields most frequently completed in the HBR are largely consistent with informal assessments reported by others [10]. In preparation for a meeting on HBRs held in Geneva, Switzerland during December 2016, field staff from John Snow, Inc., were requested to photograph (with permission) the growth monitoring charts and vaccination history recording areas in a random sample of ten HBRs for children aged <24 months in eight countries (India, Liberia, Kenya, Malawi, Mozambique, Nepal, Tanzania and Zimbabwe). In all instances, the background characteristic and vaccination history sections were consistently the most complete whereas growth monitoring charts and childhood ophthalmology were always incomplete or inadequate. In our review of Kenya HBRs of children visiting a health facility, the percentage of HBRs with markings on the growth monitoring chart was comparatively high. It is possible that the high proportion of growth monitoring charts marked is related to the non-random selection of children attending a health facility, or the result may also reflect a programmatic success in Kenya around the importance of growth monitoring among young children.

We did not assess whether recording fields were up-to-date or complete for each child or provide a comprehensive assessment of all recording fields in the Kenya maternal and child health handbook and other HBR forms. However, our observations highlight the need for a re-examination of the importance of specific content areas and recording fields included in the HBR. If recording areas are not being utilized, then programmes might consider a call to arms for the corresponding health area around the need for improvements in recording and use of data for action, or consideration should be given to removing those recording fields from the HBR altogether.

### Future research considerations

Additional work is needed to develop and operationalize effective communication strategies alongside health workers to ensure caregivers are aware of the importance of maintaining the child’s HBR safe from harm in the home and to bring the document with her to each and every health encounter. In communities where HBR loss rates exceed 10%, further steps might be taken by programmes to work with frontline health workers to understand the underlying reasons for HBR loss with follow-up corrective action(s). Additional research is needed to further understand how health workers and caregivers utilize (or not) all content areas within comprehensive, multi-domain HBRs and the benefit of these documents vis-à-vis HBRs limited to recording vaccination and those limited to vaccination and growth monitoring. Further work may also be appropriate to better understand how HBR design and standardization may influence ownership and utilization of the document, particularly with a focus on how well-designed HBRs can help reduced missed opportunities for vaccination.

## Conclusions

Opportunities exist to improve current HBR ownership levels and the understanding of the HBR as an integral component of primary health care in Kenya. The absence of a completed record of services in clear, legible handwriting compromises the function of the HBR and future healthcare delivery. Health worker failure to review the HBR as a fundamental step of each and every health encounter is one of several underlying reasons for MOV [22,23]. Improving caregiver understanding of the importance of vaccination can also facilitate a reduction in MOV.

Improving current HBR ownership levels, increasing caregiver awareness of and demand for vaccines and refining utilization by health workers are important steps towards reducing MOV and ultimately facilitating improvements in the reach of immunization services (i.e., coverage). As development partners work with the Government of Kenya towards achieving a high-performing, sustainable immunization service delivery system, we encourage inclusion of HBRs as one of the many components necessary for success.

## Supporting information

S1 Appendix Selected HBR related questions included in the Kenya MOV assessment exit interview questionnaire. (PDF)

S1 File HBR-MOV-public-use-file. (XLSX)

## Abbreviations

HBR: home-based record
MOV: missed opportunities for vaccination
WHO: World Health Organization
UNICEF: United Nations Children’s Fund
