# Supplementary material for: Home-based record (HBR) ownership and use of HBR recording fields in selected Kenyan communities: Results from the Kenya Missed Opportunities for Vaccination Assessment
Source: PLoS One. 2018 Aug 2;13(8):e0201538. doi: 10.1371/journal.pone.0201538 (PMC6072064; doi:10.1371/journal.pone.0201538)
Supplement: S1 Appendix — (PDF) [file pone.0201538.s001.pdf]

Selected home-based record related questions* included in the Kenya Missed Opportunities for Vaccination Assessment exit interview questionnaire.

- Does your child have a vaccination card / health passport?
  Response set: Yes, and I have it with me
  Yes, but I do not have it with me
  No

- Have you ever lost a vaccination card / health passport for this child?
  Response set: Yes
  No

- Did you encounter difficulty getting it replaced?
  Response set: Yes
  No

- Could you tell me what purpose the vaccination card / health passport serves?
  Response set: To know what vaccines the child has had and which ones are missing
  Other, specify
  Don't Know / No Response

- During today's clinic visit, did the staff ask you for the child's vaccination card / health passport?
  Response set: Yes
  No

- Have you ever been asked to pay for a health card / passport for your child?
  Response set: Yes
  No

- What type of health facility asked you to pay? (asked only if response 'Yes' to question above)
  Response set: Yes
  No

** Full questionnaire available from authors on request.*
